# Recent Advances in the Development of Environmentally Benign Treatments to Control Root-Knot Nematodes

**DOI:** 10.3389/fpls.2020.01125

**Published:** 2020-07-22

**Authors:** Fereidoun Forghani, Abolfazl Hajihassani

**Affiliations:** Department of Plant Pathology, University of Georgia, Tifton, GA, United States

**Keywords:** root-knot nematodes (RKNs), environmentally benign, biological control, mechanisms, emerging strategies

## Abstract

Root-knot nematodes (RKNs), *Meloidogyne* spp., are sedentary endoparasites that negatively affect almost every crop in the world. Current management practices are not enough to completely control RKN. Application of certain chemicals is also being further limited in recent years. It is therefore crucial to develop additional control strategies through the application of environmentally benign methods. There has been much research performed around the world on the topic, leading to useful outcomes and interesting findings capable of improving farmers’ income. It is important to have dependable resources gathering the data produced to facilitate future research. This review discusses recent findings on the application of environmentally benign treatments to control RKN between 2015 and April 2020. A variety of biological control strategies, natural compounds, soil amendments and other emerging strategies have been included, among which, many showed promising results in RKN control *in vitro* and/or *in vivo*. Development of these methods continues to be an area of active research, and new information on their efficacy will continuously become available. We have discussed some of the control mechanisms involved and suggestions were given on maximizing the outcome of the future efforts.

## Introduction

Nematodes are by far the most abundant animals on earth (van den Hoogen et al., 2019) and a dominant component of the soil (Bardgett and Van Der Putten, 2014). Plant-parasitic nematodes (PPNs) are a great threat to agriculture, causing an estimated annual yield loss of over $100 billion worldwide (Abad et al., 2003; Thoden et al., 2011). Among PPNs, the most yield-limiting group are root-knot nematodes (RKNs; *Meloidogyne* spp.). RKNs are obligate sedentary endoparasites which can easily reproduce in roots of over 3,000 plant species (Abad et al., 2003). They are widespread all over the world (Jones et al., 2013), and their population in the soil increases easily under appropriate conditions (Calderón-Urrea et al., 2016; Hajihassani et al., 2018).

Due to their economic importance, there is an ever-increasing need to develop sustainable management strategies and treatments for RKN control. Although cultural controls are commonly used, they are facing more limitations because of the broad host range of *Meloidogyne* spp. and the presence of mixed populations of different RKN species in the field (Trudgill and Blok, 2001; Xiang et al., 2018). The use of *Meloidogyne*-resistant cultivars has been an effective management tool for RKN; however, not many resistant cultivars are commercially available, and resistance may be overcome by new emerging RKN species such as *M. enterolobii* (Xiang et al., 2018; Hajihassani et al., 2019b).

Application of nematicides has remained the most common short-term management strategy against RKN (Hajihassani et al., 2019a; Medina-Canales et al., 2019); however, in the recent decades several chemicals such as methyl bromide and aldicarb have been withdrawn from the market due to environmental and human health concerns and toxicity to non-target organisms (Kim et al., 2018; Xiang et al., 2018). Although replacement chemicals have been developed, they have not been fully successful in gaining the same levels of efficiency (Desaeger et al., 2017). New products continue to become commercially available and are evaluated for RKN management.

Around the world, researchers have been putting efforts into developing new environmentally benign strategies for RKN management. It is important to note that results from the same treatment may vary *in vivo* and *in vitro* due to other factors in soil such as pH, organic compounds, degradation of nematicide active ingredients, chemicals and biological properties of soil, temperature, *etc.*, affecting the treatment efficiency. Plants themselves are important factors to be considered as they can have different root exudates, causing differences in the properties of their adjacent soil, namely sugars, organic acids, amino acids, microorganisms and their interactions. Root exudates may even directly affect PPN regarding their attraction and/or attachment to the plant (Bell et al., 2019). As different plants may release different chemicals into the soil as attractant/repellent agents (Sikder and Vestergård, 2020), this can be a novel opportunity to control RKN. But little is known about the specific RKN attractants, and research on this topic is at its early stages (Čepulyte et al., 2018).

Biological control such as application of live microbes (bacteria, fungi, *etc.*) and/or their secondary metabolites, essential oils, plant extracts, individual and mixed acids such as organic and amino acids, natural bioactive substances, green manure and industrial wastes are some of the environmentally benign treatments that have been studied for their efficacy against RKN. Other strategies such as ozonated water (Veronico et al., 2017), silicon (Roldi et al., 2017) and steaming and solarization (Kokalis-Burelle et al., 2016) have also been tested. In this review, we discuss the published advances on these treatments since 2015 (as of April 2020) as an all-in-one resource for RKN research.

## Biological Control

### Modes of Action

The mechanisms involved in biological control fall into two main categories: 1) antagonism against PPN and 2) plant growth promoting factors. These may result directly from the biological agents or from their metabolites (Stirling, 2014). Although other antagonists such as viruses, mites, collembola, turbellarians, oligochaetes, predaceous nematodes, and protozoans have also been tested, they have not yet been proven as efficient as bacterial or fungal organisms, or studied as much. To date, bacteria and fungi remain the prominent antagonists for PPN biocontrol (Xiang et al., 2018).

Biological agents with plant growth promoting factors are another tool considered to replace the chemicals in agriculture. These microorganisms help plants using mechanisms such as directly facilitating resource acquisition and production of cytokinin and gibberellins, and indirectly by production of antibiotics and lytic enzymes (Glick, 2012). All together, these will help the plants mitigate adverse effects of soilborne pathogens including RKN. The interaction between biological control agents with roots primes plants against RKN infection. Some of the mechanisms involved are up-regulation of several endogenous defense genes such as salicylic acid (SA)-dependent pathogenesis related genes of the systemic acquired resistance (SAR), pathogenesis related genes (*PR*-genes), *PR-1b*, *PR-1*, *PR-3*, *PR-5, ACO*, and other genes involved (Molinari and Leonetti, 2019). Enzyme activities, such as glucanase and endochitinase were also enhanced in roots of pre-treated inoculated plants. Hence, the interaction of biocontrol agents with the roots may prime plants against RKN. This topic is recently being explored and could open new doors into novel nematode control tools.

### Bacterial Microorganisms

Application of biological control agents and plant growth promotion is not a new concept (Oostendorp et al., 1991) and has already been discussed in several comprehensive reviews (Cross et al., 1999; Khan and Kim, 2007; Timper, 2014; Schouteden et al., 2015; Ntalli and Caboni, 2017; Abd-Elgawad and Askary, 2018; Xiang et al., 2018; Dutta et al., 2019). A number of different bacterial genera, namely *Pasteuria*, *Pseudomonas*, *Burkholderia*, *Arthrobacter*, *Serratia*, *Achromobacter*, and *Rhizobium* are known to have nematicidal potential.

General mechanisms of action involved in biocontrol fit into one of the categories as antibiotic production and/or other antagonism against PPN or induced resistance, which can vary even within a single genus. For example, in the genus *Bacillus*, the anti-RKN mechanism of action is based on enzymatic action for *B. firmus*, toxic antibiotics in *B. cereus* and *B. subtilis*, and toxic protein particles namely cry proteins in *B. thuringiensis* (Abd-Elgawad and Askary, 2018). Application of *B. cereus* strain BCM2 to control *M. incognita* in tomato has shown that it colonized at the root exudates and worked as a second-stage juveniles (J2) repellent, resulting in reduction of the nematode damage (Li et al., 2019). Nematode-infected tomato plants treated with BCM2 showed 67.1% fewer J2 compared with the control. In another pot study using tomato, agrobacteria were reported to enhance plant defense against RKN (Lamovšek et al., 2017). Treating the plants with *Agrobacterium tumefaciens* two days before *M. ethiopica* inoculation resulted in reduced root galling and egg counts 45 and 90 days post-inoculation (DPI). Split-root experiments showed that the observed interaction between *A. tumefaciens* and the plants was systemic.

Co-application of *Chitiniphilus* sp. strain MTN22 and *Streptomyces* sp. MTN14 on Brahmi (*Bacopa monnieri*) has been reported to mitigate the *M. incognita* mediated oxidative stress and augment the bacoside content of the plant (Gupta et al., 2017). In greenhouse studies, fresh and dry biomass were improved by 1.7 and 2.1-fold respectively, upon treatment. Improvements were confirmed using electron microscopy through examination of J2 and eggs, and nematode numbers in the roots. A study has shown that a commercial biocontrol product (NemOut^TM^) consisted of *B. subtilis*, *B. licheniformis*, and *Trichoderma longibrachiatum* could inhibit *M. incognita* reproduction on tomato (Silva J. de O. et al., 2017). The optimal dose (10 kg/ha) reduced *M. incognita* population density up to 56.5–63.9% compared to the control 65 DPI in the greenhouse. However, application of the fungus *Pochonia chlamydosporia* was still more efficient. None of these microorganisms had negative impacts on tomato growth.

*Bacillus amyloliquefaciens strain* Y1 was evaluated to control *M. incognita in vitro* and *in vivo* on tomato. Bacterial culture supernatant and crude extract of Y1 significantly inhibited the hatching of RKN eggs and caused J2 mortality (Jamal et al., 2017). The 10–40% supernatant (10–40 µl of bacterial supernatant adjusted to final volume of 100 µl using sterile distilled water) concentrations inhibited egg hatching by 32.5–60.6% after five days of exposure *in vitro*. The J2 mortality increased with increasing treatment concentration and exposure time, reaching the maximum (80%) after three days at 40% concentration. Plant growth parameters were significantly higher in the Y1-treated plants compared to the untreated controls. The researchers were also able to identify a dipeptide cyclo (D-Pro-L-Leu) compound as the anti-RKN compound using chromatographic techniques and nuclear magnetic resonance.

Another study aiming to control *M. incognita* in tomato was performed using *B. cereus* strain Jdm1 (Xiao et al., 2018). Culture supernatant significantly inhibited the egg hatching and reduced J2 numbers *in vivo*. Additionally, Jdm1 treatment decreased the root galling severity (43%) and promoted tomato growth performance. Field studies showed a greater control efficacy of up to 50% for gall index 30 DPI. Bacterial community of the tomato rhizosphere was initially impacted upon treatment, but soon recovered.

A commercial *B. firmus-*based product (Flocter; Bayer CropScience) alone and in combination with synthetic nematicides oxamyl (Vydate; Corteva Agriscience) and fosthiazate (Nemathorin; Syngenta) was examined under greenhouse conditions for the control of *M. incognita* in two independent cropping cycles of tomato (d’Errico et al., 2019). Application of *B. firmus* either alone or in combination with nematicides suppressed nematode population levels in the second crop cycle. However, combined application of *B. firmus* and nematicides resulted in lower galling severity. Root galling index (scale of 0–10) was 7.7 for the control while it was 5.9, 5.5, 5.8, 4.3, and 4.0 for *B. firmus*, oxamyl, fosthiazate, *B. firmus* + oxamyl, and *B. firmus* + fosthiazate, respectively. Thus, the application of *B. firmus* early in the growing season would be effective for the management of *M. incognita* on tomato. Most of these studies were focused on the application of a single strain. It would be promising to test whether co-application of the same bacterial genera members would result in better protection of roots against RKN and to determine if possible synergism exists among the closely related bacteria helping them to better dominate the soil microbiome.

Successful application of *B. subtilis*, *B. pumilus*, and *P. fluorescens* against *M. incognita* on cowpea in a greenhouse study has also been reported (El-Nagdi et al., 2019). The best reduction in nematode numbers was achieved using *P. fluorescens* (89%) followed by combined treatments of *P. fluorescens* and *B. subtilis* (88.50%). The highest yield increase (70.2%) was recorded in combined treatment, followed by 49.3% by *B. pumilus*. Successful application of *Bacillus* for RKN management has also been reported elsewhere, either alone or in combination with *Streptomyces rubrogriseus* and the nematicide Fosthiazate (Yue et al., 2019). These biocontrol agents are promising, but their activity should be evaluated under field conditions.

Another example of *Bacillus* with great anti-RKN potential is *B. pumilus* strain L1. Lee and Kim (2016) reported that this strain produced both protease and chitinase enzymes, which were the main causes for its *M. arenaria* antagonistic characteristics. The 10% bacterial culture caused 88% inhibition of egg hatching and approximately 90% J2 mortality in comparison to the control. Tomato pot experiments revealed significant reduction in the number of galls and egg masses as well as J2 population in soil six weeks after *M. arenaria* infestation. *B. pumilus* L1 not only could be a potential biocontrol agent for RKN but also caused plant growth promotion. Different *Bacillus* may possess different anti-RKN mechanisms, creating numerous paths of research to explore by combining these strains.

*Pasteuria penetrans*, a widely distributed endospore forming bacterium is well known as a RKN parasite (Liu et al., 2017). Its mechanisms of action are mainly based on affecting the RKN J2. The J2 infected with a few spores of *P. penetrans* will later produce a few or no eggs in host plants, whereas with increasing the number of spores, the J2 becomes less mobile and its ability to penetrate roots can be reduced (Chen and Dickson, 1998; Liu et al., 2017). In a recent work mainly focusing on the physicochemical and biological properties of coffee rhizosphere, the researchers wanted to assess how such properties would influence the *M. exigua* suppression in the field. In the majority of the coffee farms with highest suppressive effects on RKN, *P. penetrans* was a major component of the soil rhizosphere (Botelho et al., 2019). In suppressive soils, approximately 83% of J2s were dead resulting in the highest coffee bean yields.

Soil microbiome was evaluated for *M. hapla* and *M. incognita* suppression (Topalovic et al., 2019). Four soil series with similar texture, organic matter content and pH were collected from different geographical locations. However, only one of the soils was identified as a potential RKN-suppressive soil, and its microbiome was harvested and inoculated into tomato pots infested with *M. hapla* and *M. incognita*. In the case of *M. hapla*, the RKN-suppressive soil had 32% fewer galls per root than the other soils tested. The suppressive soil was less efficacious in controlling *M. incognita* compared to *M. hapla.* Interestingly, microbial suspensions from the suppressive soil did not affect mortality of any of the nematode species tested *in vitro*, in contrast to the pot studies, highlighting the fact that plant-mediated antagonism and other factors in the soil may play a vital role in the anti-RKN properties of biocontrol strategies.

Rhizobacteria improve soil texture, and the compounds they secrete constitute valuable biostimulants modulating plants’ stress responses. These interactions are managed via signal exchanges between plant roots and the microbes (Ma et al., 2011; Backer et al., 2018). It has been proven that biological growth promoting factors may improve plant growth and reduce the negative effect of RKN in seedlings (Khanna et al., 2019). In that study, the effect of plant growth promoting bacteria (*Pseudomonas aeruginosa* and *Burkholderia gladioli*) on growth and antioxidative potential in nematode-infected tomato was assessed, and microorganisms with growth promoting characteristics improved tomato defense to encounter oxidative stress generated under RKN infection. A similar study using tomato infected with *M. incognita* reported that each of *B. cereus*, *B. licheniformis*, and *P. fluorescens* significantly reduced J2 numbers, and plants with *B. cereus* had lower root galling compared to other treatments (Colagiero et al., 2018).

Viljoen et al. (2019) screened 27 plant growth promoting rhizobacteria strains against *M. incognita* on 6-week-old carrot seedlings. Five of the strains, *B. firmus* T11, *B. aryabhattai* A08, *Paenibacillus barcinonensis* A10, *P. alvei* T30, and *B. cereus* N10w caused 86, 85, 85, 81, and 82% reduction in gall numbers, respectively. In the greenhouse, *P. alvei* T30 and *B. aryabhattai* A08 showed potential as biological control agents of *M. incognita* on carrot and tomato, respectively.

### Fungal Biocontrol

Application of fungal microorganisms for managing RKN as well as other agricultural pests has been ongoing for decades. Some of the most prominent fungal species for RKN control belong to *Actylellina*, *Arthrobotrys*, *Aspergillus*, *Catenaria*, *Dactylellina*, *Hirsutella*, *Pochonia*, *Purpureocillium*, and *Trichoderma* (Timper, 2014; Abd-Elgawad and Askary, 2018). *Arthrobotrys* and *Dactylellina* can trap RKN J2s in the soil using their hyphal *structures* and decrease the invasion ability of the nematode. However, the extent of RKN mitigation may be influenced by several factors, as soil is a dynamic matrix (Saxena et al., 1987; Hsueh et al., 2013; Wang et al., 2014).

Colonization of plants by particular endophytic fungi can provide plants improved defense against PPN. The mechanisms involved seem to be multifactorial (Schouten, 2016), which are found within a range of genera such as *Acremonium*, *Alternaria*, *Fusarium*, and *Trichoderma* (Qiang et al., 2012). These mechanisms may include repellent activity reducing J2 attraction to roots (Le et al., 2016), attenuated or delayed development of adult females and reduction of their fecundity (Martinuz et al., 2013). Some endophytes such as *Paecilomyces* and *Trichoderma* may also trap and kill RKN in the soil or root systems. These may act at different nematode life stages (*i.e.* eggs, juveniles, or adults) (Bordallo et al., 2002; Rumbos and Kiewnick, 2006; Yao et al., 2015; Schouten, 2016). Competition for resources such as sugars is another mechanism used (Hofmann et al., 2009a; Hofmann et al., 2009b). Production and secretion of nematicidal metabolites have also been confirmed by several *in vitro* and *in vivo* studies (Bush et al., 1993; Kunkel and Grewal, 2003; Goswami et al., 2008; Bacetty et al., 2009; Tian et al., 2014). Finally, production of plant like hormones such as cytokinins and gibberellins and mobilizing and enhancing plant defense are two other mechanisms that some endophytic fungi use (van Loon et al., 1998; Rodriguez et al., 2009; Redman et al., 2011; Sikder and Vestergård, 2020). A novel study recently reported that *P. chlamydosporia* can induce plant-dependent systemic resistance to *M. incognita* (Ghahremani et al., 2019).

Arbuscular mycorrhizal fungi (AMF) are obligate root symbionts that help protect their host plants against biotic stresses such as PPN infection. This is achieved via a number of mechanisms such as higher nutrient uptake, altered root morphology, competing for space and nutrition with PPN, and inducing plant systemic resistance (Schouteden et al., 2015). The host provides photosynthetic carbon for AMF (Gianinazzi et al., 2010) and AMF help with the uptake of water, minerals and even micro-elements (Smith and Smith, 2011; Baum et al., 2015). The symbiosis may also result in increased root growth and branching (Vos et al., 2014). Overexpression of pathogenicity-related genes in plants due to AMF symbiosis has also been reported (Andrade et al., 2010; Hao et al., 2012).

*Meloidogyne enterolobii* is an emerging and destructive RKN species, and there is ongoing research on the characterization and/or development of biological microorganisms for its control (Silva S. D. et al., 2017). The inhibiting effects of two egg-parasitic fungi, *Pochonia chlamydosporia* (19 strains) and *Purpureocillium* *lilacinum* (14 strains) against *M. enterolobii* were assessed on agar plates. Reductions of 13 to 84% were observed depending on the fungal strain used. Combining fungal strains, however, did not improve the anti-hatching effect. The most efficacious strains of *P. chlamydosporia* (CG1006, CG1044) and *P. lilacinum* (CG1042, CG1101) were selected for further experiments. Future research should focus on assessing the potential of application timing of biological microorganisms on RKN control and targeting better development of fungal hyphae or conidial structures. Results of the pot studies using tomato and banana revealed that optimal fungal treatments were able to reduce post-harvest egg numbers of RKN in the plants inoculated with 500 eggs. The control effect was mitigated as initial nematode inoculation level increased. In one experiment for banana, the number of eggs after 12 months of *P. chlamydosporia* inoculation was reduced by 34%, and no significant reduction was observed in tomato plants after 3 months. In another experiment with tomato using *P. chlamydosporia* and *P. lilacinum*, the number of eggs was reduced by 34 and 44%, respectively, when initial infestation level was low (500 eggs). It was concluded that the application of *P. chlamydosporia* and *P. lilacinum* could be used as a component of an integrated pest management (IPM) approach in fields with low pressure of the nematode.

In contrast, the effect of three *P. chlamydospora* isolates (Pc-3, Pc-10, and Pc-28) for the control of *M. javanica* on tomato was not promising (Xavier et al., 2017) as none of the isolates were able to reduce gall severity whether applied individually or in combination. However, the number of eggs was decreased in comparison to the control for isolate Pc-10 and the mixtures Pc-10 + Pc-3 and Pc-10 + Pc28 + Pc-3. In another combined study, suitability of *P. chlamydosporia* for RKN management was further confirmed (Escudero et al., 2017). The novelty of this work was the co-application of chitosan and *P. chlamydosporia* resulting in increased root colonization by the fungi and accordingly higher reductions in RKN damage.

The effect of *P. lilacinum* strain PL251 combined with fluopyram was tested against *M. incognita* (Dahlin et al., 2019). In the greenhouse, the commercial nematicide Velum and strain PL251 were formulated as a wettable granular and applied on tomato. Reductions of 56 and 68% in the number of J2 were achieved after PL251 being used alone and in combination with Velum, respectively. A similar reduction in the root galling was observed for which the index (scale of 0–10) was 3.8 for the control *versus* 1.8 for the plants with the combined treatment. The researchers suggested that Velum would reduce the nematode population at planting and therefore reinforce the biocontrol efficacy of *P. lilacinum* during the growing season.

Kim et al. (2018) screened 500 isolates of endolichenic fungi, *Xylaria* sp., and found that only one strain “KCTC 13121” had strong nematicidal activity. The *in vitro* studies revealed that grammicin at concentrations of 15.9 and 5.87 µg/ml was able to control *M. incognita* with 50% efficiency against J2 and egg hatching after 3 and 14 days, respectively. In addition, the wettable powder-type formulation and fermentation broth filtrate from *X. grammica* KCTC 13121 were able to suppress the development of RKN in tomato and melon. The entomopatogenic fungus *Lecanicillium muscarium*, relatively known as a biocontrol agent against insect pests (Faria and Wraight, 2007), was studied against *M. incognita* in both *in vitro* and *in vivo* conditions (Hussain et al., 2018). Three *L. muscarium* isolates (Lm1, Lm2, Lm3) were effective against *M. incognita* with Lm1 being prominent. Tomato treated with *L. muscarium* was positively affected for plant growth compared with control. Only 5% of the fungus-infected eggs hatched *in vitro* in contrast to 96% in the control. Greenhouse studies also showed that Lm1 decreased the number of galls and eggs by 80 and 90%, respectively.

Another fungus recently studied for its anti-RKN effects was *Metarhizium guizhouense* PSUM02 (Thongkaewyuan and Chairin, 2018). Crude extracts of the fungus grown on a protein-enriched medium contained a protease (33 kDa) which in combination with the fungus spores (1 × 10^4^ spores/ml) completely inhibited hatching of the *M. incognita* eggs within 24 h *in vitro*. The mortality of J2 increased to 100%, 48 h post-treatment. The mycelia invaded immature eggs, while the eggs containing juveniles and the hatched juveniles were resistant. Electron microscopy observations showed extensive damage to eggshell and cuticle upon individual and combined treatments.

*Trichoderma longibrachiatum* was evaluated *in vitro* for its biocontrol efficacy against *M. incognita* (Zhang et al., 2015). Results showed a strong lethal effect (>88%) on the nematode when J2s were exposed for 14 days to 1 × 10^5^ to 1 × 10^7^ conidia/ml. The same concentrations of the fungus significantly reduced *M. incognita* infection in cucumber and improved plant growth in the greenhouse. *Trichoderma* strains have also been proven effective both as plant growth promoter and *M. incognita* control agent on pepper (Herrera-Parra et al., 2017). Approximately, 22 to 35% reductions in galling index were reported in pots treated with *T. atroviride*, *T. virens*, and *T. harzianum*-C2. In addition, *T. atroviride* reduced the nematode egg production by 63% and the number of females by 14.36%.

Effect of *Trichoderma* spp. and *P. lilacinum* on *M. javanica* in a commercial pineapple production setting has also been studied (Kiriga et al., 2018). Three *Trichoderma* isolates, *T. asperellum* M_2_RT_4_, *T. atroviride* F_5_S_21_, and *Trichoderma* sp. MK_4_ and two *P. lilacinum* (KLF_2_ and MR_2_) were used. Using individual inocula, *T. asperellum* M_2_RT_4_ and MK_4_ as well as the two *P. lilacinum* isolates reduced *M. javanica* root galling from 61 to 82%. *Trichoderma asperellum* M_2_RT_4_ was the most effective fungus reducing galling, egg mass and egg numbers by over 82, 78, and 88%, respectively. It also increased root fresh weight by 91%.

*Trichoderma* spp. may reduce RKN infections through triggering host defense. A group of researchers investigated whether *Trichoderma* modulates the hormone signaling network in the host to induce resistance to nematodes (Martínez-Medina et al., 2017). Using *M. incognita*, they found that root colonization by *Trichoderma* prevented nematode performance both locally and systemically at multiple stages such as invasion, gall formation and reproduction. First, *Trichoderma* primed SA-regulated defenses, limiting nematode root invasion. Then, it enhanced jasmonic acid (JA) regulated defenses, thereby antagonizing the deregulation of JA-dependent immunity by the nematodes, compromising galling and fecundity.

One of the most recent species characterized as anti-RKN is *Mortierella globalpina* (DiLegge et al., 2019). *Caenorhabditis elegans* was infected with a soil slurry containing a microbiome likely to house nematophagous microbes. Infected nematodes were sub-cultured repeatedly to isolate pure cultures of the microbe with nematicidal activity. Pure cultures were confirmed to be antagonistic to *C. elegans* and were identified as *M. globalpina*. *In vitro* studies showed that *M. globalpina* trapped *M*. *chitwoodi via* hyphal adhesion to its cuticle layer, penetrated it and killed the nematode by digesting its cellular contents. The effectiveness of fungi on both eggs and J2–J4 stages was confirmed using electron microscopy. In the *in vivo* experiments, *M*. *globalpina* promoted the plant growth. It would be crucial to test this approach on other major RKN species including *M. incognita* and *M. arenaria* to assess its potential broad-spectrum effect. Another path to take would be combining this species with other microbes in search of developing a dynamic multi-dimensional biological control inoculum for agricultural application.

The possibility of using mushrooms as anti-nematode tools has been gaining attraction. In this context, recently the ovicidal, nematicidal, and nematistatic potential of crude extract and metabolites retained from liquid mushroom cultures was assessed against *M. javanica* (Hahn et al., 2019). The interaction of the mushroom mycelium and *M. javanica* J2 was also examined. A total of 24 mushroom isolates from 15 different species were included. Among them, *Lentinula edodes*, *Macrocybe titans*, and *Pleurotus eryngii* reduced the nematode damage in all experiments. Mushrooms have therefore the potential to control RKN.

**Table 1** shows a summary of the biocontrol methods and strategies evaluated since 2015. In comparison to the conventional chemicals, these methods typically need a longer time to show visible results against RKN. Also, their efficacy may drop in the field compared to controlled experimental conditions, which can be due to various external factors in soil impacting the microorganisms. Therefore, it is necessary to put more effort into developing improved biocontrol strategies. One of the possibilities can be the application of targeted/specialized methods. It would be suitable to have the soil properties data such as biological, chemical and texture factors and choose the best biological agent with the highest adjustability to that particular soil.

**Table 1:** Summary of biological control strategies tested from 2015 to April 2020 for the control of root-knot nematodes (*Meloidogyne* spp.).

Organism	RKN species	Type of study	Host plant	Reference
Bacteria				
*Bacillus cereus*	*M. incognita* *M. incognita* *M. incognita*	*In vitro, in vivo* *In vitro, in vivo* *in vitro, in vivo*	Tomato Tomato Tomato	(Li et al., 2019) (Xiao et al., 2018) (Colagiero et al., 2018)
*Agrobactrium tumefaciens*	*M. ethiopica*	*In vivo*	Tomato	(Lamovšek et al., 2017)
*Bacillus amiloliquefaciens*	*M. incognita*	*In vitro, in vivo*	Tomato	(Jamal et al., 2017)
*Bacillus firmus*	*M. incognita*	*In vivo*	Tomato	(d’Errico et al., 2019)
*Pseudomonas fluorescens*	*M. incognita* *M. incognita*	*In vivo* *In vitro, in vivo*	Cowpea Tomato	(El-Nagdi et al., 2019) (Colagiero et al., 2018)
*Bacillus pumilus*	*M. arenaria*	*In vitro, in vivo*	Tomato	(Lee and Kim, 2016)
*Bacillus licheniformis*	*M. incognita*	*In vitro, in vivo*	Tomato	(Colagiero et al., 2018)
*Pasteuria penetranse*	*M. exigua*	*In vivo*	Coffee	(Botelho et al., 2019)
**Fungi**				
*Pochonia chlamydosporia*	*M. incognita* *M. enterolobii*	*In vivo* *In vitro, in vivo*	Tomato Banana/Tomato	(Silva J. de O. et al., 2017) (Silva S. D. et al., 2017)
*Purpureocillium lilacinum*	*M. enterolobii* *M. javanica*	*In vitro, in vivo* *In vivo*	Banana/Tomato Pineapple	(Silva S. D. et al., 2017) (Kiriga et al., 2018)
*Xylaria grammica*	*M. incognita*	*In vitro, in vivo*	Melon/Tomato	(Kim et al., 2018)
*Lecanicillium muscarium*	*M. incognita*	*In vitro, in vivo*	Tomato	(Hussain et al., 2018)
*Metarhizium guizhouense*	*M. incognita*	*In vitro*	–	(Thongkaewyuan and Chairin, 2018)
*Trichoderma longibrachiatum*	*M. incognita*	*In vitro, in vivo*	Cucumber	(Zhang et al., 2015)
*Trichoderma atroviride* *Virens harzianum*	*M. incognita*	*In vivo*	Pepper	(Herrera-Parra et al., 2017)
*Trichoderma asperellum*	*M. javanica*	*In vivo*	Pineapple	(Kiriga et al., 2018)
*Mortierella globalpina*	*M. chitwoodi*	*In vitro, in vivo*	Pepper	(DiLegge et al., 2019)
**Mushrooms**				
*Lentinula edodes* *Macrocybe titans* *Pleurotus eryngii*	*M. javanica*	*In vitro*	–	(Hahn et al., 2019)
**Co-application**				
*Pochonia chlamydosporia* & Chitosan	*M. javanica*	*In vitro, in vivo*	Tomato	(Escudero et al., 2017)
*Chitiniphilus* sp. & *Streptomyces* sp.	*M. incognita*	*In vivo*	Brahmi	(Gupta et al., 2017)
*Pseudomonas fluorescens* & *Bacillus subtilis*	*M. incognita*	*In vivo*	Cowpea	(El-Nagdi et al., 2019)
*Bacillus subtilis*, *Trichoderma longibrachiatum*, & *Bacillus licheniformis*	*M. incognita*	*In vivo*	Tomato	(Silva J. de O. et al., 2017)
Soil microbiome	*M. incognita*, *M. hapla*	*In vivo*	Tomato	(Topalovic et al., 2019)
*Purpureocillium lilacinum* & Nematicide “Velum”	*M. incognita*	*In vivo*	Tomato	(Dahlin et al., 2019)

## Natural Compounds

### Application of Acids

A variety of different organic acids including amino, acetic, butyric, formic, and propionic acids are known to have toxic effects on certain species of PPN. These are the result of either microbial decomposition of different compounds in the soil or metabolites produced by microorganisms (Oka, 2010). Several successful applications of acids against nematodes have been reported such as heptalic acid (Abd-Elgawad and Askary, 2018) and hydroxamic acids (Zasada et al., 2005). However, acid’s ability against nematodes is heavily influenced by soil conditions (Momma et al., 2006). In a study conducted *in vitro* and in the greenhouse, the nematicidal efficacy of 5-aminolevulinic acid (ALA) on *M. incognita* and other PPN was tested (Cheng et al., 2017) in which ALA exhibited a strong anti-RKN effect and reduced egg hatching of *M. incognita* by 90%. Treatment of the soil with 6.0 mM ALA reduced the galling index (scale of 0–4) from 2.3 in control to approximately 0.3 on tomato. The number of egg masses per root was decreased from about 180 to only 50. It also significantly altered the nematode metabolism including the total protein production, malondialdehyde content, and oxidase activities, suggesting that ALA is a promising biodegradable bionematicide.

In a study on plant secondary metabolites, the anti-RKN capacity of acetic acid and other natural compounds was evaluated against *M. incognita* (Ntalli et al., 2016). The acid treated J2s were examined by electron microscopy, showing that acetic acid harmed the J2 cuticle and degenerated the nuclei of pseudocoel cells and vacuolized the cytoplasm resulting in nematode death. In another work, a total of 237 bacterial strains were screened for their nematicidal activity against RKN. Among those, *Lactobacillus brevis* strain WiKim0069 isolated from kimchi, a Korean fermented food, was found to produce organic acids with nematicidal activity (Seo et al., 2019). The culture filtrate of WiKim0069 had a pH of 4.2 and contained acetic acid (11,190 μg/ml), lactic acid (7,790 μg/ml), malic acid (470 μg/ml), and succinic acid (660 μg/ml). An artificial mixture of the four organic acids produced by WiKim0069 also induced 98% *M. incognita* J2 mortality at a concentration of 1.25%. When the filtrate was tested on *M. incognita* infested plants in pot experiments, it suppressed the formation of galls and egg masses on tomato roots in a dose dependent manner. It was also capable of reducing galls on melon by 62.80% in the field study. We have discussed this study here and not in the biological control section, as such entitlement would be more suitable in case WiKim0069 can produce similar promising results upon application to the soil.

Humic acid was evaluated to control *M. incognita* infecting banana (Seenivasan and Senthilnathan, 2018). *In vitro*, humic acid at 0.08 to 2.0% concentration inhibited egg hatching between 50 and 100%. In parallel, it reduced the mobility of J2 in a concentration affected manner. Pot experiments showed that soil humic acid treatment reduced root galling. Despite the humic acid concentrations used (ranging from 0.04 to 0.4%), the nematode density reduction in soil was 53.5–56.7%, and egg population reduction was 61.9–63.8%. Plant growth improvement was another advantage of humic acid treatment. The positive effect of humic acid on PPN control however, still needs to be confirmed by evaluating other crops under field conditions.

### Application of Oils

Scientists and industry have been evaluating the potential of essential oils (EOs) as biopesticides for RKN control. EOs were evaluated as soil biofumigants for the control of *M. incognita* on tomato in greenhouse (Laquale et al., 2015). Three different concentrations (50, 100, and 200 µl/kg soil) of EOs from five different plants were tested. EOs of *Eucalyptus globulus* and *Pelargonium asperum* significantly reduced nematode multiplication and gall formation on roots at all concentrations and increased top and root biomass. In another study, 29 plant-based EOs were evaluated against *M. incognita* both *in vitro* and *in vivo* on tomato (Barros et al., 2019a). Fourteen EOs showed nematicidal activities of 8 to 100% at a concentration of 1,000 µg/ml. The most prominent activity was from Mexican tea (*Dysphania ambrosioides*) EO, which reduced the number of galls and eggs by 99.5 and 100%, respectively. The researchers performed further analysis using chromatography-mass spectrometry and detected (Z)-ascaridole (87.28%), E-ascaridole (8.45%), and *p-cymene* (3.35%), representing 99.08% of the total oil composition from *D. ambrosioides*.

Oils from three Brazilian plants (*Astronium graveolens*, *Hyptis suaveolens*, and *Piptadenia viridiflora*) were evaluated against *M. incognita* both *in vitro* and *in vivo* (Barros et al., 2019b). Only *P.* *viridiflora* showed toxicity against *M. incognita* and was further studied. *P.* *viridiflora* major component, benzaldehyde, was identified using the gas chromatography–mass spectrometry and tested against *M. incognita*. It was able to produce up to 65% reduction in the number of eggs while its oxime compound was capable of reducing both galls (up to 84%) and eggs (up to 89%) on tomato. Another work studied the activity of EO extracted from high and low land plants of *Artemisia nilagrica* against *M. incognita* (Kalaiselvi et al., 2019). The altitude at which the plants were grown in affected the nature, quantity, appearance, yield, and chemical composition of the EOs produced. The lethal concentrations (LCs) were also different at 5.75 and 10.23 µg/ml (LC_50/48h_) for EOs extracted from high and low altitude plants, respectively. Both EOs reduced root infection by *M. incognita* on tomato and promoted plant growth under greenhouse conditions. Nematode population (eggs and J2) in the root (10 g) was reduced for about 87 and 68%, following treatment by EOs from high- and low-level plants, respectively. Exposing *C. elegans* as a model organism to *A. nilagrica* oils enhanced its intracellular reactive oxygen species (ROS) production and germline cell apoptosis.

### Plant Extracts and Compounds

The nematicidal activity of *Camellia oleifera* and *Paenoia rockii* extracts against *M. incognita* was tested *in vitro* by Wen et al. (2019). The extracts from *C. oleifera* cake and *P. rockii* stems suppressed egg hatching. These extracts also had a nematotoxic activity on J2, with *P. rockii* being superior. At 5 mg/ml, the *P. rockii* extract caused 100% inactivation of *M. incognita* J2 after seven days. In another study, plant extracts from root and shoot of vetiver (*Vetiveria zizanioides*), a known nonhost grass for certain nematodes, were both toxic on *M. incognita*, resulting in 40 and 70% J2 mortality, respectively (Jindapunnapat et al., 2018). The plant extracts were also repellent to J2 and contained a combination of oil and acid. In a greenhouse study using water extracts from fruits of *Melia azedarach* (Chinaberry), researchers reported success in the control of *M. incognita* and *M. javanica* and increase in soil biological activity (Ntalli et al., 2018). On tomato, *M. incognita* females decreased from about 69 in one gram of roots to only 16 for the extract-treated plants. A similar trend was observed for *M. javanica* with the numbers dropping from 79 to 4.2. In the field studies, the number of J2 were about 3,564 for the untreated control compared to about 1,056 for the water-extract treated plants. Microbial properties of the soils included in the study remained the same, while the frequency of free-living nematodes was increased gradually.

The inhibitory effect of allicin (diallyl thiosulfinate), the key natural antimicrobial compound of garlic was evaluated against *M. incognita * (Ji et al., 2019). *In vitro* studies showed that allicin effectively inhibited the nematode J2. The median LC_50_ was 18.23 mg/L. In the greenhouse experiments, allicin controlled *M. incognita* and improved tomato yield. More interestingly, it increased superoxide dismutase, catalase and peroxidase activity in tomato leaves compared to the untreated control. Thus, allicin could be a potential substitute to synthetic chemical compounds for the control of RKN. For future, it is important to assess if production of an extract would be economically advantageous in large scale for agricultural application. In addition, the activity of these compounds (*e.g.* sorption, movement, or degradation) in the soil under field conditions should be evaluated.

The effect of bioactive saponins extracted from five *Medicago* spp. was evaluated *in vitro* against a number of PPN including *M. incognita* (D’Addabbo et al., 2020). Overall, the efficacy of saponins varied depending on the plant species used. At 500 µg/ml, J2 mortality was over 90% after 8–16 h contact time, while at 1,000 µg/ml, egg hatching ranged from 18 to 39% compared to the water control. Results from this study demonstrate that saponin-rich extracts and plant biomasses from *M. heyniana*, *M. hybrida*, *M. lupulina*, *M. murex*, and *M. truncatula* can be highly suppressive to RKN. Care should be taken in future studies to include toxicity tests assuring the safety of optimal nematicidal dosages of saponin against non-target organisms as they have been reported to be occasionally dangerous to embryonic cells of vertebrates (Hassan et al., 2008).

Plant defense proteins “lectins” are a heterogeneous group of bioactive proteins or glycoproteins which can selectively be recognized and bonded to specific glycans in a reversible manner (Peumans and Van Damme, 1995). *Abelmoschus esculentus* lectin (AEL) was characterized on the basis of structural insights and found to be toxic on *M. incognita* and *M. javanica* (de Lacerda et al., 2017). The AEL (500 µg/ml) reduced J2 hatching with greater effect observed for *M. incognita*. The number of *M. incognita* J2 hatched after 9 days of contact to AEL was about 120 compared to about 220 for the control. In the case of *M. javanica*, the numbers were about 60 and 90 for treated samples and control, respectively. This work expanded the knowledge on application of lectins as part of RKN management strategies, highlighting the importance of their deeper study to identify new suitable lectins and the mechanisms involved.

## Other Strategies

### Soil Modifications

Although using organic soil amendments is a conventional method for managing PPN in different cropping systems (Oka, 2010), there are still emerging amendments being developed. In this context, a green manure derived from *Fumaria parviflora* was evaluated as a soil amendment to control *M. incognita* in tomato under greenhouse and field conditions (Naz et al., 2015). Potted soil was amended with fresh chopped whole plant material of *F. parviflora* at rates of 0 (control), 10, 20, and 30 g/kg, 15 days prior to transplanting, and then 15 and 30 days afterwards. The best control of *M. incognita* resulted when plants were treated with 30 g/kg amendment. The galling index was about 4 (scale of 0–5) for the untreated control in contrast to 1.04 for treated samples. The number of J2s per cm^3^ of soil were 414 and 127 for the control and treated plants, respectively. In addition, the root portion of *F. parviflora* showed higher nematicidal activity than the aboveground parts.

The bioactivity of chitin oligosaccharide (COS) dithiocarbamate derivatives against *M. incognita* was evaluated in a study (Fan et al., 2019), in which three different COS derivatives were synthesized and their structures were characterized. Subsequently, nematicidal and egg hatching inhibitory effects, plant growth promotion, toxicity, and phytotoxicity were assessed. As of the mechanism of action, they inferred that nematicidal activity may be correlated with glutathione-binding activity but not heavy metal ion complexation. Two out of three COS derivatives tested were able to reduce egg hatching for over 60% when the concentrations exceeded 0.5 mg/ml. At 2 mg/ml, the 1, 3-dithicyclobutane-N-chitosan oligosaccharide (COSDTB) derivative was able to reduce hatching up to 90%. It also caused 94% J2 mortality at 4 mg/ml. Upon COS treatment, metabolization of tomato plants increased, and photosynthetic pigment contents of leaves were elevated with low phytotoxicity damage. The application of MCF-7 cells in cultures treated with 3 mg/ml COS derivatives resulted in more than 90% viability, demonstrating low cytotoxicity towards mammalian cells. Future studies should be expanded in terms of their inclusiveness for different *Meloidogyne* species, host crops, and soil types.

Silicon’s ability to control *M. paranaensis* mediated by coffee plants was evaluated (Roldi et al., 2017). Coffee seedlings were treated with silicate before the inoculation of *M. paranaensis* in contrast to the non-treated control. After transplanting, 2,000 eggs were inoculated to each plant and sampling and data collection was performed periodically. Root microscopic observations confirmed a reduction in J2 penetration upon silicon treatment. In the two independent experiments performed, silicon was able to reduce the number of *M. paranaensis* per g of root as much as 89%. Treatment in the form of potassium silicate did not affect plant growth.

### Soil Treatments

Soil solarization has been used as a pest management technique for decades and was studied against RKN as well (Nico et al., 2003; Melero-Vara et al., 2012). Since 2015, soil solarization and its combination with soil steaming were investigated to develop an affordable, yet efficient approach for the management of RKN in floriculture crops including larkspur, snapdragon, and sunflowers (Kokalis-Burelle et al., 2016). The nematicide methyl bromide was used for comparison. Root galling severity on all three crops was lower in steam treatments than in solarization alone. Steam treatment also resulted in the control of *M. arenaria* comparable to, or greater than that of methyl bromide. In conclusion, it was suggested that steaming followed by solarization would be an efficient method to replace harmful chemicals in floriculture.

Ozonated water (O_3_wat) is a well-known treatment in agriculture that inactivates pathogens. It has recently been proven suitable for plant irrigation without causing negative effects (Martínez-Sánchez and Aguayo, 2020). In search of new anti-RKN treatments, the ability of O_3_wat to control *M. incognita* was evaluated (Veronico et al., 2017), showing that O_3_wat helped protect tomato against *M. incognita* through the modulation of basal defense mechanisms. There was no phytotoxicity damage due to the application of O_3_wat on tomato. The root galling index (scale of 0–10) in O_3_wat-treated plants was significantly lower than that of untreated control. Untreated control, O_3_wat treated samples after nematode inoculation and O_3_wat treated samples before nematode inoculation had a root galling index of 3.9, 1.9, and 1.6, respectively. Measuring ROS and H_2_O_2_ in galls showed their elevated production upon O_3_wat treatment. The positive effect would probably be related to the modulated antioxidant systems, which increase the ROS, H_2_O_2_ and malondialdehyde in nematode feeding sites. Due to its nature, O_3_wat will degrade to water within a short time, giving the treatment several advantages for RKN management. For example, O_3_wat treatment can be performed as an early treatment in an integrated RKN management approach without affecting soil properties.

### Application of Industrial Wastes

With global industrialization and mass production, it is necessary to find new ways of turning industrial wastes into value added products. There have been very few attempts until now to use this concept for RKN management. In one of these studies, agro-industrial wastes, namely rice husk, common bean hull, soybean hull, orange bagasse, poultry litter and a mixture of equal portions of these materials were evaluated against *M. javanica* using pot studies in the greenhouse (Brito et al., 2020). In brief, powdered bean hulls, soybean hulls, orange bagasse and waste mixtures were the most efficient wastes with RKN control ranging from 55 to 100%.

Manipueira is the common name of a liquid residue discharged from cassava (*Manihot esculenta*) starch factories that is rich in nutrients and cyanogenic glycosides. Its nematicidal effect against *M. incognita* was evaluated in a tomato field (Nasu et al., 2015). At 50% concentration, Manipueria showed nematicidal and plant growth promotion activity. Sisal, a native plant of Mexico is becoming more popular in parts of the world as a source of fiber. The effect of sisal liquid residue (fresh and fermented) derived from its industrial processing was evaluated against *M. javanica* (Damasceno et al., 2015). A mortality rate of 100% was obtained for J2 exposed to liquid residue at a concentration of 20% *in vitro*. In the greenhouse it also reduced the number of galls and egg masses per gram of tomato roots, as well as the final population of *M. javanica* in the soil. The fermented liquid residue caused inhibition of the beneficial microorganisms, in contrast to the fresh liquid residue.

## Conclusions and Prospects

Considering the importance of economic losses caused by RKN and the fact that the restrictions governing the use of chemical nematicides are elevating, it is clear that there is need for the development of new environmentally benign strategies. It is also crucial to continue improving the current green methods in search of making them more efficient. For example, some questions to be addressed for antagonistic fungi/bacteria include what is the optimum rate, timing, frequency and method of application for biocontrol agents especially under field conditions? In addition, agricultural industry and scientists should focus on keeping these developed and/or optimized methods economically advantageous, so that they can be adopted by growers on different farming scales. Biocontrol of RKN has been around for decades, but it is still capable of achieving much more attention and better results as new species are identified, characterized and evaluated for their efficacy against RKN. Currently, targeted sequencing such as 16S and 18S rDNA sequencing has great potential to be applied for detection of new biological agents in RKN management. This will make the biocontrol studies faster, cheaper, and more practical. Furthermore, it would be helpful to focus on the microbiomes of RKN suppressive soils based on the meta data in the future studies, in order to explore possibilities for developing more holistic management strategies with multi-target modes of action.

To date, environmentally benign methods are mostly not fully competent on their own with the traditional chemical practices in terms of protecting plants against RKN. Therefore, it is critical to consider the development and improvement of multidisciplinary management strategies for RKN such as combining microbial strategies using both bacterial and fungal agents with other cultural control practices or host resistance. Both biocontrol and application of soil amendments have been studied to some extent against PPN, but with the recent advances in technology there is room for deeper studies on how these two strategies can be synergistically used. For example, studies on how the application of certain amendments may influence the soil microbiome in relation to nematodes inhibition. More tools such as O_3_wat are also becoming available which may be incorporated into the multi-aspect strategies developed.

In conclusion, future studies should focus on environmentally benign approaches which are based on multidisciplinary strategies that can fill the gaps of single sided management methods. Such approaches will also reduce the chance of resistance as the complexity of different nematicidal components would make resistance highly improbable. Whatever strategy is devised, future attempts should focus on important factors such as synergism between RKN antagonists, environmental conditions, sustainability, studying the effect of new treatments on non-target organisms, and association of individual plants with nematode antagonists of interest.

## Author Contributions

FF designed the project. Both authors participated in writing and editing the manuscript.

## Conflict of Interest

The authors declare that the research was conducted in the absence of any commercial or financial relationships that could be construed as a potential conflict of interest.
